# Per- and Polyfluoroalkyl Substance-Induced Skin Barrier Disruption and the Potential Role of Calcitriol in Atopic Dermatitis

**DOI:** 10.3390/ijms26157085

**Published:** 2025-07-23

**Authors:** JinKyeong Kim, SoYeon Yu, JeongHyeop Choo, HyeonYeong Lee, Seung Yong Hwang

**Affiliations:** 1 Department of Molecular & Life Science, Hanyang University, Sangnok-gu, Ansan 15588, Gyeonggi-do, Republic of Korea; jink0207@naver.com (J.K.); cnwjdguq@naver.com (J.C.); nick7904@naver.com (H.L.); 2 Institute of Science and Convergence Technology, Hanyang University, Sangnok-gu, Ansan 15588, Gyeonggi-do, Republic of Korea; yusso3027@naver.com; 3 Major in Molecular Medicine, School of Biopharmaceutical Convergence, College of Advanced Technology and Convergence, Hanyang University Sangnok-gu, Ansan 15588, Gyeonggi-do, Republic of Korea

**Keywords:** atopic dermatitis, per- and polyfluoroalkyl substances, calcitriol, gene expression

## Abstract

Environmental exposure to per- and polyfluoroalkyl substances (PFASs) has been increasingly associated with skin disorders, including atopic dermatitis (AD); however, the underlying molecular mechanisms remain unclear. This study aimed to evaluate the effects of perfluorononanoic acid (PFNA) and perfluorooctanoic acid (PFOA)—two widely detected PFASs—on epidermal function and gene expression in Human Epithelial Keratinocyte, neonatal (HEKn). We assessed cell viability, morphology, and transcriptomic changes using in vitro assays and RNA-seq analysis from a neonatal cohort. PFASs induced dose-dependent cytotoxicity and downregulation of barrier-related genes. Ingenuity pathway analysis identified calcitriol as a suppressed upstream regulator. Functional validation revealed that calcitriol partially reversed the PFAS-induced suppression of antimicrobial peptide genes. These findings support the hypothesis that PFASs may contribute to AD-like skin pathology by impairing vitamin D receptor signaling and antimicrobial defense, and calcitriol demonstrates potential as a protective modulator. This study provides mechanistic insights into the impact of environmental toxicants on skin homeostasis and suggests a potential protective role for calcitriol in PFAS-induced skin barrier damage.

## 1. Introduction

Atopic dermatitis (AD) is a common inflammatory disease that typically develops during childhood and is characterized by chronically dry, pruritic, and eczematous skin lesions [1]. AD is triggered by complex interactions between genetic predispositions and environmental factors, affecting up to 20% of children and 10% of adults worldwide [2,3]. In recent years, increasing levels of environmental pollutants associated with urbanization and industrialization have been identified as key risk factors for AD [4]. Consequently, a growing body of research has investigated the relationship between AD and various environmental toxicants, including particulate matter (PM), volatile organic compounds (VOCs), heavy metals, and per- and polyfluoroalkyl substances (PFASs) [5].

The onset of AD is typically attributed to the interplay between epidermal barrier dysfunction and immune dysregulation. Terminally differentiated keratinocytes express various barrier-related proteins. In particular, the granular layer contains essential structural proteins such as filaggrin (FLG), loricrin (LOR), and involucrin (IVL), as well as tight junction proteins, including claudins, occludin, and zonula occludens (ZO) proteins. Abnormal expression of these proteins compromises the skin barrier, leading to increased transepidermal water loss (TEWL) and enhanced permeability to allergens, irritants, and pathogens [6]. This subsequently promotes immune dysregulation via the secretion of Th2-type cytokines such as interleukin IL-4 and IL-13. In addition, the synthesis of key antimicrobial peptides (AMPs) such as cathelicidins and defensins is often suppressed. These interrelated factors create a vicious cycle that exacerbates AD pathogenesis [7].

Per- and polyfluoroalkyl substances (PFASs) are a group of synthetic chemicals containing fluorinated carbon chains. Due to their oil- and water-repellent properties, thermal stability, chemical resistance, and surfactant activity, PFASs have been widely used in household products, carpets, clothing, and food packaging [8]. However, PFASs are highly persistent in the environment and accumulate in air, water, and wildlife [9]. In humans, they exhibit long biological half-lives (approximately 3–5 years), and have been associated with adverse effects on immune, developmental, reproductive, and neurological systems [10]. These characteristics have led to their classification as “forever chemicals,” highlighting their potential health risks [11]. Among them, perfluorooctanesulfonic acid (PFOS) and perfluorooctanoic acid (PFOA) have been banned by the Stockholm Convention due to their toxicity. As substitutes, other long-chain PFASs such as perfluorononanoic acid (PFNA), a nine-carbon perfluorocarboxylic acid (PFCA), have garnered interest. Although structurally similar to the eight-carbon PFOA, PFNA may differ in its toxicological profiles and biological effects [12,13].

Epidemiological studies have suggested a modest positive association between PFAS exposure and the development of AD. Chronic exposure to PFASs may modulate immune responses, potentially increasing AD risk. Notably, several studies have reported that higher serum perfluorononanoic acid (PFNA) levels in adolescents are significantly associated with increased AD prevalence [14]. In a Taiwanese birth cohort, maternal exposure to PFOA was positively correlated with early-onset AD in children at age five [15]. Additional cohort studies have demonstrated significant associations between prenatal exposure to PFOA or PFNA and AD incidence in early childhood, particularly among female offspring [9]. Furthermore, another study reported a positive correlation between prenatal PFOA exposure and AD onset by age two, especially in children with deletions of glutathione S-transferase (*GSTT1* or *GSTM1*), suggesting that genetic susceptibility may modulate individual responses to PFAS exposure [16]. These studies suggest that PFASs may contribute to the pathogenesis of AD through mechanisms involving immune dysregulation, oxidative stress, and impairment of epidermal barrier function. While previous research has predominantly explored PFAS toxicity using epidemiological approaches, in vitro studies at the cellular level remain limited.

In this study, we aimed to evaluate the toxic effects of PFASs in the Human Epithelial Keratinocyte, neonatal (HEKn) cell line and examine the resulting changes in gene expression at the mRNA level to assess their potential impact on AD pathogenesis. The central focus of the study was the examination of the cellular toxicity of PFASs in human keratinocytes. Additionally, we aimed to integrate in vitro data with transcriptomic results from a neonatal birth cohort, thereby bridging epidemiological and mechanistic insights.

## 2. Results

### 2.1. PFAS Exposure Reduces HEKn Cell Viability in a Dose-Dependent Manner

**Figure 1:**
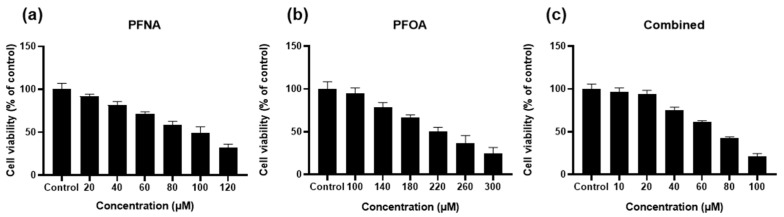
Dose-dependent effect of PFASs on HEKn cell viability. HEKn cells were treated for 24 h with a range of concentrations of (**a**) PFNA, (**b**)PFOA, or (**c**) a combination of both compounds at equal concentrations (Combined). Cell viability was assessed using the MTS assay and expressed as relative absorbance compared to the untreated control. Results are presented as mean ± SD from at least three independent experiments performed in sextuplicate. Statistical significance: *p* < 0.05.

To evaluate the cytotoxicity effects of PFASs, HEKn cells were treated with PFNA, PFOA, or a combined mixture of both compounds (Combined), followed by an MTS assay. After 24 h of exposure, all treatment groups exhibited a dose-dependent decrease in cell viability (Figure 1). Among the three groups, PFOA alone showed the least cytotoxicity, whereas the Combined treatment resulted in the greatest reduction in viability, suggesting a potential synergistic effect between the two compounds. Based on these results, the inhibitory concentrations (IC values) for each treatment were calculated. We selected the IC_25_ concentration for subsequent experiments. Selected IC values are in Table 1.

### 2.2. PFAS Exposure Alters Cell Morphology and Induces Cell Death

**Figure 2:**
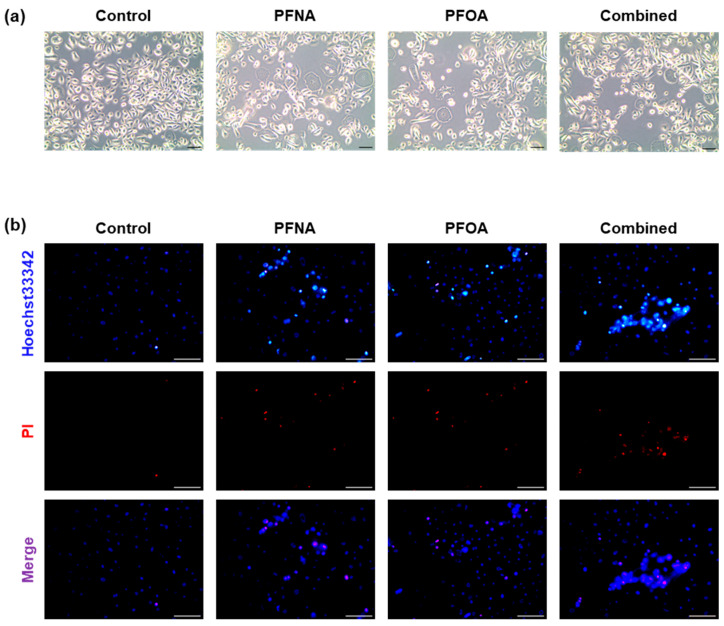
Morphological changes and cell death in HEKn cells following PFAS exposure. (**a**) Representative phase-contrast images (10× magnification) of HEKn cells treated with PFNA, PFOA, or a combined mixture at their respective IC_25_ concentrations for 24 h. (**b**) Apoptosis was assessed using Hoechst 33342/PI double staining and observed under fluorescence microscopy (20× magnification). Hoechst 33342 stained all nuclei blue, whereas PI specifically stained the nuclei of cells with damaged membranes in red. Images are representative of at least three independent experiments. (Scale bar = 100 μm).

To visually assess PFAS-induced morphological changes and cell death in HEKn, cells were treated with PFNA, PFOA, or a combined mixture (Combined) at their respective IC_25_ concentrations for 24 h. Phase-contrast microscopy revealed distinct differences in cell morphology across treatment groups (Figure 2a). Untreated control cells exhibited high cell density and preserved cellular morphology. In contrast, PFAS-treated cells showed reduced confluency and increased intercellular spacing, and some cells displayed shrinkage or irregular shapes.

To further evaluate PFAS-induced cytotoxicity, Hoechst 33342/propidium iodide (PI) double staining was performed (Figure 2b). Hoechst 33342 stains all nuclei blue, whereas PI selectively penetrates cells with compromised membranes, labeling apoptotic or necrotic nuclei with red fluorescence [17]. In the control group, most cells were Hoechst^+^/PI^−^, indicating intact membranes. In contrast, PFNA- and PFOA-treated groups displayed PI-positive cells, and the number of PI-positive nuclei was highest in the combined group. These results suggest that PFAS exposure compromises membrane integrity and promotes cell death in HEKn cells, consistent with the cytotoxicity result observed in the MTS assay.

### 2.3. PFASs Disrupt Epidermal Barrier-Related Gene Expression in HEKn

**Figure 3:**
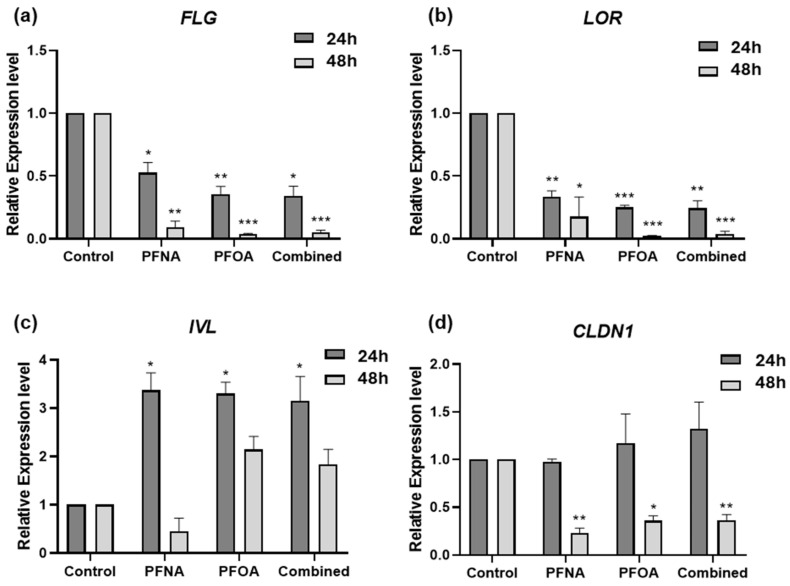
Alterations in the expression of skin barrier-related genes following PFAS exposure. HEKn cells were treated with PFNA, PFOA, or Combined at their IC_25_ concentrations for 24 and 48 h. The mRNA levels of (**a**) *FLG*, (**b**) *LOR*, (**c**) *IVL*, and (**d**) *CLDN1* were measured by RT-qPCR. Gene expression was normalized to *GAPDH* and expressed relative to untreated controls. Data are presented as mean ± SEM from at least three independent experiments, each performed in triplicate. Statistical significance was evaluated using Student’s *t*-test: * *p* < 0.05, ** *p* < 0.01, *** *p* < 0.001.

To assess the effects of PFNA, PFOA, and their combination (Combined) on the expression of skin barrier-related genes, differentiated HEKn cells were treated with each compound at their respective IC_25_ concentrations for 24 and 48 h. The mRNA levels of *filaggrin (FLG)*, *loricrin (LOR)*, *involucrin (IVL)*, and *claudin-1 (CLDN1)* were quantified by RT-qPCR using *GAPDH* as the reference gene. All data were normalized to untreated controls (Figure 3).

In PFAS-treated HEKn cells, *FLG* expression was significantly downregulated at both 24 and 48 h (Figure 3a), with a time-dependent decrease observed in all treatment groups. *LOR* was significantly downregulated at both time points (Figure 3b), with a more marked reduction at 48 h. In contrast, *IVL* expression was significantly upregulated at 24 h in all PFAS groups (Figure 3c), and while its expression declined slightly at 48 h, it remained elevated compared to controls. This upregulation may reflect a compensatory mechanism in response to barrier disruption caused by reduced *FLG* and *LOR* expression, as has been reported in barrier-defective skin models [18]. *CLDN1* expression remained unchanged at 24 h in all treatment groups (Figure 3d). However, by 48 h, significant downregulation was observed in all PFAS-treated cells, suggesting a delayed but notable disruption of tight junction integrity.

### 2.4. PFNA and PFOA Exposure Induces Differential Expression of AD-Related Genes

To investigate transcriptomic alterations associated with PFAS exposure in the context of AD, RNA-seq-based differential gene expression (DEG) analysis was performed. Based on serum PFAS concentrations measured from 116 neonates, participants were stratified into high (H)- and low (L)-exposure groups for both PFNA and PFOA, corresponding to the upper and lower 30% of concentrations, respectively. Each exposure group was further subdivided into atopic (A) and non-atopic (NA) individuals based on clinical AD diagnosis, resulting in a total of eight subgroups. Detailed subgroup characteristics are summarized in Table 2. The average serum concentrations were 0.778 μg/L for PFNA-H, 0.339 μg/L for PFNA-L, 4.279 μg/L for PFOA-H, and 1.487 μg/L for PFOA-L, with PFOA levels being generally higher than those of PFNA.

**Figure 4:**
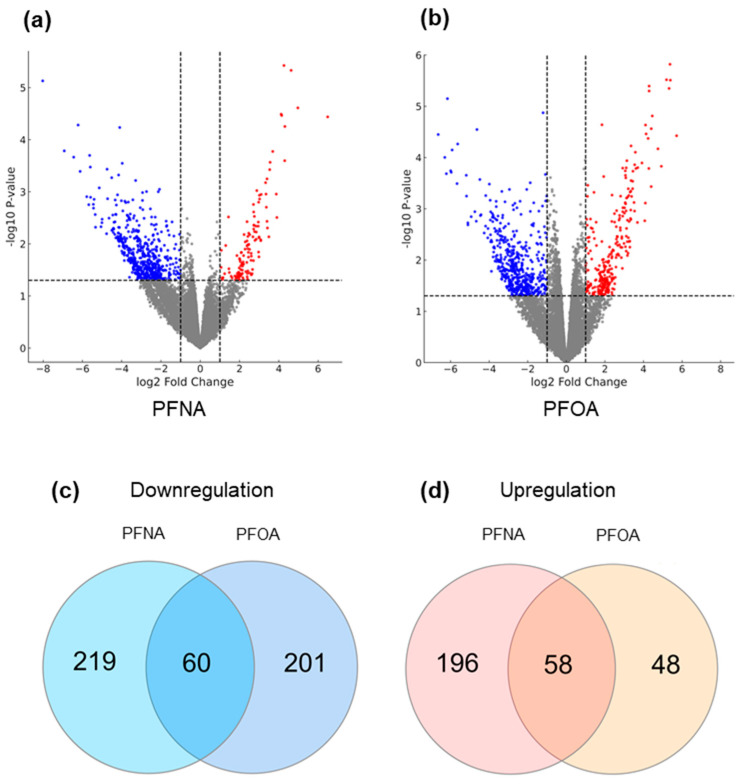
Differential gene expression and shared gene sets under high PFAS exposure and AD conditions. (**a**) DEG analysis was conducted by comparing the high-exposure atopic group to the low-exposure non-atopic group for (**a**) PFNA and (**b**) PFOA. Volcano plots display significantly altered genes. Dashed lines represent the cutoffs for significance (log_2_ fold change = ±1 and *p* = 0.05), and the grey area indicates non-significant genes. Upregulated and downregulated genes are shown in red and blue, respectively. To isolate genes uniquely influenced by both PFAS exposure and AD status, additional DEG comparisons with the low-exposure atopic and high-exposure non-atopic groups were performed, and overlapping genes were excluded. The remaining genes were analyzed by Venn diagrams to identify (**c**) downregulated genes and (**d**) upregulated genes in both PFNA and PFOA groups.

To assess the combined effects of PFAS exposure and AD status, we designated the low-exposure non-atopic group as the reference control and compared it to the high-exposure atopic group for each PFAS. DEG analysis identified 822 and 665 significantly DEGs for PFNA and PFOA, respectively (|log_2_ fold change| > 1, *p*-value < 0.05), as visualized by volcano plots (Figure 4a,b). In both datasets, the number of downregulated genes exceeded that of upregulated genes, and PFNA yielded a greater number of DEGs overall.

To refine the gene sets specifically affected by the interaction of PFAS exposure and AD, additional DEG analyses were conducted comparing the low-exposure atopic group and the high-exposure non-atopic group against the reference. Genes with overlapping expression changes (in both direction and significance) across these comparisons were excluded. As a result, 44 upregulated and 245 downregulated genes were removed from the PFNA dataset, yielding a refined list of 533 PFNA-specific DEGs. Similarly, 18 upregulated and 280 downregulated genes were excluded from the PFOA dataset, resulting in 367 PFOA-specific DEGs. Venn diagram analysis revealed that 58 upregulated and 60 downregulated genes were commonly altered in both PFNA and PFOA datasets (Figure 4c,d). These findings suggest that, although PFNA and PFOA may act through distinct biological pathways, their structural similarity may allow them to converge on a shared set of transcriptional targets. The full lists of DEGs under PFNA and PFOA exposure conditions are provided in Supplementary Tables S1 and S2.

### 2.5. Calcitriol Functions as an Upstream Regulator of PFNA- and PFOA-Associated Gene Expression in AD

**Figure 5:**
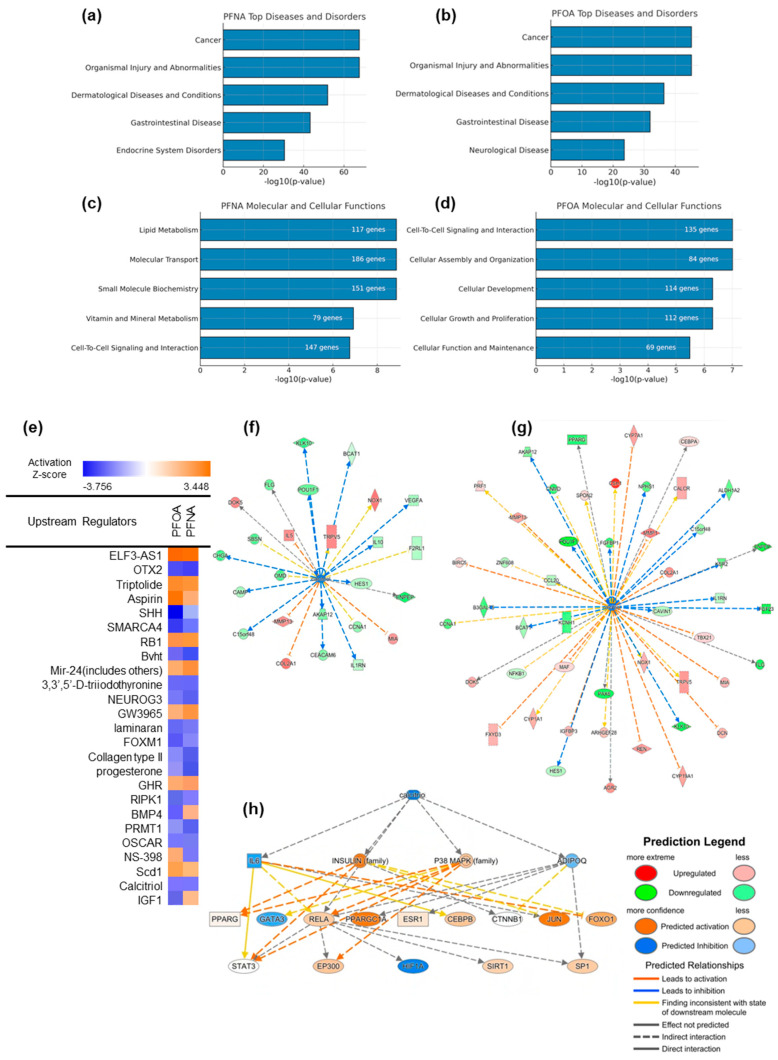
Ingenuity Pathway Analysis (IPA) of PFNA- and PFOA-induced transcriptomic changes under atopic dermatitis conditions. (**a**,**b**) Top enriched categories in the “Diseases and Disorders” domain for PFNA and PFOA, respectively. (**c**,**d**) Top molecular and cellular functions affected by each PFAS, ranked by –log_10_(*p*-value). (**e**) Predicted upstream regulators for PFNA and PFOA exposures, visualized based on z-scores indicating predicted activation or inhibition. (**f**,**g**) Gene networks of calcitriol-regulated downstream molecules, filtered from DEG datasets of PFNA- and PFOA-exposed atopic groups. (**h**) A broader upstream interaction network centered on calcitriol and its key downstream regulators, inferred from the IPA knowledge base. Node shapes indicate molecular categories defined by IPA.

To explore the biological pathways and upstream regulators modulated by PFAS exposure, we performed Ingenuity Pathway Analysis (IPA) based on the results of DEG analysis. In the “Diseases and Disorders” category, both PFNA and PFOA exposures were associated with enrichment in dermatological diseases and conditions, as well as cancer and organismal injury (Figure 5a,b), suggesting that PFAS exposure may contribute to AD through diverse pathological mechanisms.

In the “Molecular and Cellular Functions” category, PFNA exposure affected pathways related to lipid metabolism, cell-to-cell signaling, and small molecule biochemistry—processes that are closely linked to epidermal differentiation and metabolic homeostasis (Figure 5c). In contrast, PFOA exposure was more strongly associated with cellular growth, development, and organization (Figure 5d), indicating that although PFNA and PFOA share some mechanistic overlap, they may contribute to AD pathogenesis via distinct biological routes.

Upstream regulator analysis predicted multiple transcriptional regulators that may drive the observed gene expression changes under PFNA and PFOA exposure (Figure 5e). Some regulators were activated or inhibited in both PFAS conditions, while others exhibited compound-specific regulatory patterns. Calcitriol was significantly inhibited according to the upstream regulator analysis. Considering the known link between vitamin D signaling and atopic dermatitis, additional analyses were conducted to investigate this pathway.

To visualize the interaction between PFASs and the calcitriol pathway, we constructed a gene network comprising known calcitriol target genes whose expression was significantly altered in our DEG dataset (Figure 5f,g). The strong concordance between predicted IPA targets and our experimental data supports the hypothesis that PFAS exposure may suppress calcitriol-mediated signaling. Furthermore, calcitriol was identified as a top-tier upstream regulator influencing a broader regulatory network relevant to AD pathogenesis (Figure 5h). Major signaling nodes such as *IL6*, *RELA*, *STAT3*, *PPARGC1A*, *FOXO1*, *JUN*, and *GATA3* were predicted to be modulated, either activated or inhibited, under PFAS exposure. Among these, the *IL6–STAT3* axis is a well-established driver of chronic inflammation and was predicted to be indirectly regulated by calcitriol [19]. This network structure suggests that PFAS-induced suppression of calcitriol may disrupt immune homeostasis and contribute to sustained skin barrier dysfunction and inflammation. The complete list of downstream molecules regulated by calcitriol is presented in Supplementary Table S3.

### 2.6. Calcitriol Modulates the Expression of Antimicrobial Peptide Genes in AD

**Figure 6:**
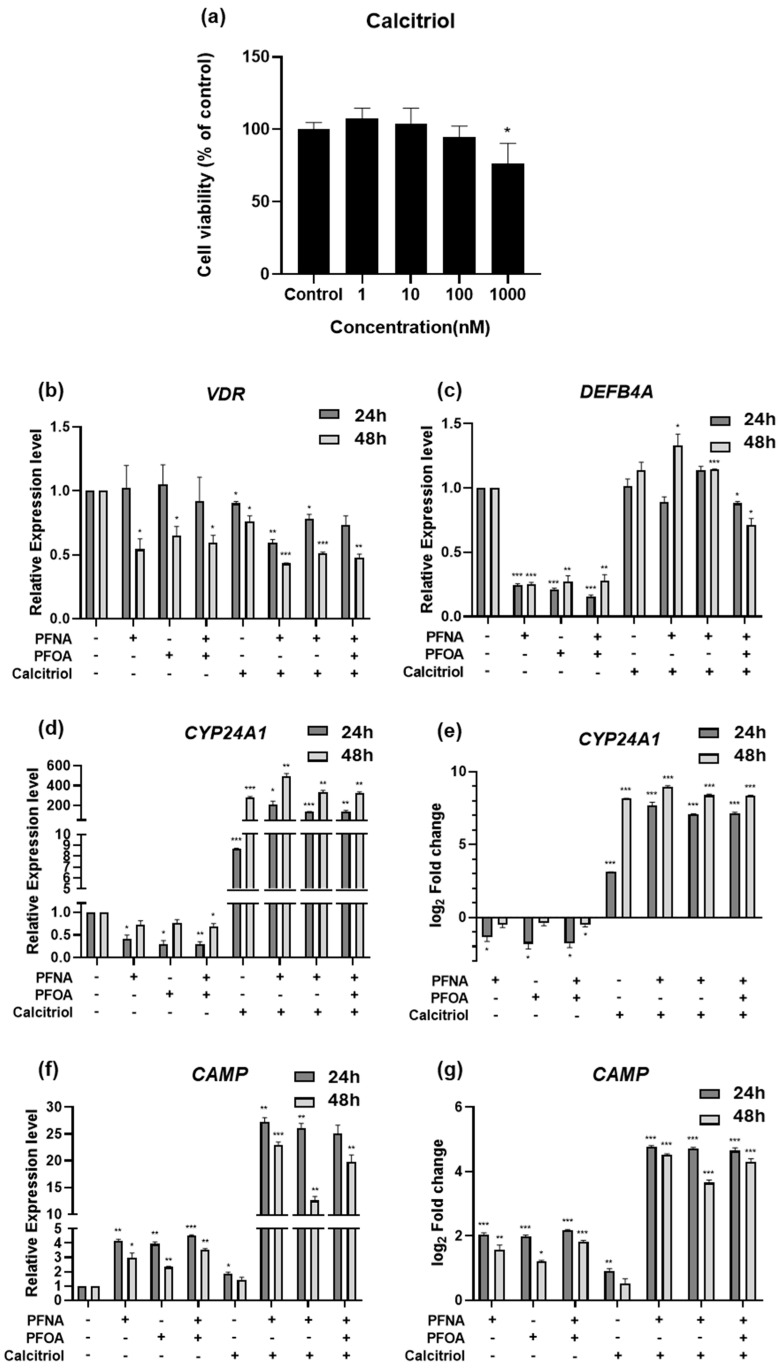
Gene expression changes following treatment with calcitriol alone or in combination with PFASs in HEKn. (**a**) Cell viability was assessed by MTS assay after 24 h treatment with increasing concentrations of calcitriol. HEKn cells were treated with PFASs, calcitriol, or a combination for 24 or 48 h. Results are presented as mean ± SD from at least three independent experiments performed in sextuplicate. The mRNA expression levels of calcitriol-related genes, including (**b**) *VDR*, (**c**) *DEFB4A*, (**d**,**e**) *CYP24A1*, and (**f**,**g**) *CAMP*, were quantified by RT-qPCR. Expression values were normalized to *GAPDH* and presented as relative expression or log_2_ fold change compared to untreated controls. Data represent mean ± SEM from at least three independent experiments, each performed in triplicate. Statistical significance was determined using Student’s *t*-test: * *p* < 0.05, ** *p* < 0.01, *** *p* < 0.001.

To examine the functional role of calcitriol under PFAS exposure, HEKn cells were treated with PFNA, PFOA, or both in combination with calcitriol, and gene expression was analyzed by RT-qPCR using *GAPDH* as the reference gene. First, we assessed the cytotoxicity of calcitriol using an MTS assay. No significant changes in cell viability were observed across concentrations ranging from 1 to 100 nM (Figure 6a), and thus 10 nM—where no cytotoxic effects were detected—was selected for subsequent experiments.

The expression of *VDR*, the receptor for calcitriol, remained unchanged at 24 h post-treatment (Figure 6b). However, after 48 h of PFAS exposure, *VDR* expression was significantly downregulated, and this trend persisted in the calcitriol co-treatment group, suggesting that PFASs may suppress vitamin D signaling via inhibition of VDR expression. *CYP24A1* was slightly downregulated upon PFAS exposure, with a more noticeable decrease at 24 h, followed by partial recovery at 48 h (Figure 6d,e). In contrast, calcitriol alone led to a marked increase in *CYP24A1* expression, particularly at 48 h. Notably, *CYP24A1* expression was significantly higher in the calcitriol + PFAS co-treatment group compared to that in the calcitriol alone group, suggesting a potential synergistic effect between PFASs and calcitriol.

To investigate whether this interaction influences immune barrier function, we assessed the mRNA expression of antimicrobial peptide genes *Defensin Beta 4A (DEFB4A)* and *Cathelicidin Antimicrobial Peptide (CAMP)*, both known to be inducible by calcitriol [20,21]. *DEFB4A* was downregulated to a similar extent by PFAS exposure at both 24 and 48 h (Figure 6c). Calcitriol alone did not significantly alter *DEFB4A* expression, but co-treatment with PFASs restored its expression to levels comparable to those of the untreated control. Interestingly, *CAMP* expression was elevated by PFAS exposure alone (Figure 6f,g), indicating a potential stress- or inflammation-induced response. However, calcitriol alone did not strongly induce *CAMP*, and this may suggest that PFASs create an inflammatory milieu that sensitizes cells to calcitriol-induced responses. In the co-treatment group, *CAMP* expression was significantly higher than in either treatment alone, reinforcing the hypothesis of a synergistic interaction between PFASs and calcitriol. Similar to *CYP24A1*, this pattern suggests that calcitriol may demonstrate enhanced transcriptional activity in the presence of PFAS-induced stress.

## 3. Discussion

Humans are simultaneously exposed to a mixture of PFASs through various consumer products and environmental sources [22]. In line with this concern, PFOS was classified as a persistent organic pollutant (POP) under the Stockholm Convention in 2009 and is currently regulated in 152 signatory countries. The European Union also regulates the use, intake, and environmental levels of PFOS, PFOA, PFNA, and PFHxS [23]. This study aimed to investigate the potential contribution of PFASs to the pathogenesis of AD. We demonstrated that PFNA, PFOA, and a combination significantly reduced HEKn cell viability in a dose-dependent manner and induced morphological alterations and cell death. Notably, the combined treatment exhibited a stronger cytotoxic effect, suggesting possible synergistic interactions between multiple PFASs.

Considering that the IC_50_ values for PFASs were substantially higher than typical environmental exposure levels and could lead to significant cell death [24], we selected the IC_25_ concentration for subsequent experiments. This decision was based on the understanding that environmental toxicants are usually encountered at low concentrations through chronic exposure [25], and the chosen dose should be sufficient to induce transcriptional changes without causing overt cytotoxicity. Although the IC_25_ concentration used in this study is higher than typical PFAS levels observed in human serum, it represents a suitable experimental threshold for inducing early cellular responses. Therefore, this concentration allows for molecular analysis under non-cytotoxic conditions.

*FLG* encodes a large structural protein essential for cornified envelope formation, skin hydration, and antimicrobial defense. Loss-of-function mutations or reduced expression of *FLG* are strongly associated with early-onset and persistent AD, as well as enhanced allergic sensitization [26]. *LOR* and *IVL* are both members of the epidermal differentiation complex (EDC) and contribute to cornified envelope integrity [27]. *CLDN1*, a critical tight junction protein involved in epidermal differentiation and intercellular adhesion, is commonly downregulated in both lesional and non-lesional skin of AD patients [28]. RT-qPCR analysis revealed that key skin barrier genes—*FLG, LOR*, and *CLDN1*—were downregulated following PFAS exposure, whereas *IVL* showed compensatory upregulation. This may reflect differences in the temporal expression patterns of these genes during keratinocyte differentiation, where *IVL* is considered an early differentiation marker and *FLG* and *LOR* are late markers [29]. *CLDN1* was particularly downregulated after 48 h, suggesting a time-dependent disruption of tight junctions by PFAS exposure. These findings support the hypothesis that PFASs can impair skin barrier integrity through temporally and concentration-dependent transcriptional regulation of multiple barrier-associated genes.

Our RNA-seq analysis based on a neonatal cohort identified a significant number of DEGs in the high-exposure AD groups for both PFNA and PFOA. While some genes were commonly altered by both compounds, others showed distinct expression patterns. Structurally, PFASs are composed of a carbon backbone with fluorine atom substitutions and terminal functional groups [30]. They are classified as perfluoroalkyl substances when all hydrogen atoms are replaced by fluorine, and polyfluoroalkyl substances when only some substitutions occur. Both PFNA and PFOA are perfluoroalkyl substances with carboxylic acid (–COOH) functional groups. The main structural difference lies in the length of their carbon chains—PFNA has nine carbons, while PFOA has eight [31]. Generally, longer carbon chains correlate with greater bioaccumulation potential, which may explain the distinct biological effects observed despite their structural similarity [32].

Low serum vitamin D levels are associated with increased AD prevalence and severity [33]. Topical calcitriol has been shown to restore epidermal permeability and antimicrobial barrier function, and improve skin barrier integrity in NC/Nga mice [6,34]. Notably, recent in silico modeling studies suggest that PFASs exhibit high binding affinity to VDR, potentially interfering with the calcitriol–VDR interaction and downstream signaling [35]. IPA identified calcitriol as a suppressed upstream regulator in both PFNA- and PFOA-exposed AD conditions. This highlights the potential role of the calcitriol–VDR signaling axis in AD pathophysiology. *Cytochrome P450 Family 24 Subfamily A Member 1 (CYP24A1)* encodes a mitochondrial cytochrome P450 enzyme that inactivates calcitriol and is transcriptionally induced by calcitriol in most tissues, thus serving as a feedback regulator [36]. To experimentally validate this finding, we treated HEKn cells with PFAS and calcitriol and examined the expression of *VDR* and its downstream targets—*CYP24A1, DEFB4A*, and *CAMP*. These genes encode key components of the chemical barrier that protect against microbial invasion and contribute to cytokine-mediated barrier reinforcement and wound healing [37]. While calcitriol alone had low effects on *DEFB4A* and *CAMP* expression, co-treatment with PFAS restored *DEFB4A* levels and significantly enhanced *CAMP* expression. This finding is consistent with previous reports that calcitriol enhances *DEFB4A* expression under inflammatory conditions, suggesting that calcitriol may counteract PFAS-induced repression of antimicrobial genes during inflammation [38]. Additionally, the observed induction of *CYP24A1* under co-treatment conditions supports the notion of a synergistic interaction between PFASs and calcitriol [39]. These results imply that PFASs may impair skin barrier integrity and reduce antimicrobial defense by disrupting the calcitriol–VDR signaling pathway, contributing to AD exacerbation. While our findings highlight the ability of calcitriol to reverse PFAS-induced suppression of antimicrobial peptide genes, it remains unclear whether these effects are specific to PFAS-induced inflammation or reflect a broader anti-inflammatory and barrier-enhancing role of calcitriol. Previous studies have shown that calcitriol can restore antimicrobial peptide expression suppressed by topical corticosteroid treatment [34]. Furthermore, the presence of vitamin D response element (VDRE) in the promoter region of the *CAMP* [40] supports the notion that calcitriol may exert a general immunomodulatory effect, independent of the specific type of inflammatory stimuli. These findings suggest that the beneficial effects of calcitriol may extend beyond PFAS exposure and contribute more broadly to skin barrier restoration under inflammatory conditions.

A key limitation of this study is the use of HEKn cells as a mono-culture model, which does not account for interactions with immune cells. To partially address this, we also analyzed RNA-seq data from peripheral blood mononuclear cells obtained from the same cohort. Future studies should incorporate co-culture models with immune cells or cytokine treatments like IL-4 and IL-13 to better simulate the AD microenvironment. In addition, three-dimensional skin organoid models could offer more physiologically relevant insights. For a more accurate assessment of environmental relevance, long-term studies addressing chronic exposure and accumulation of PFASs are also necessary. In addition, our in vitro findings support a potential inhibitory interaction between PFASs and the VDR–CYP24A1 axis; however, direct evidence demonstrating changes in VDR nuclear translocation or ligand binding following PFAS exposure is currently lacking. Future studies employing nuclear localization analysis or VDR-binding reporter assays would be valuable for validating this mechanistic association.

In this study, we aimed to explore the potential interaction between PFAS exposure and atopic status through stratified analysis; however, these two variables may not be entirely independent within the study population. Therefore, the possibility of residual confounding by unmeasured factors—such as socioeconomic status, dietary habits, or genetic background—cannot be fully excluded. To more accurately disentangle the effects of PFAS exposure, future population-based studies incorporating a broader range of contextual and environmental variables are warranted.

In summary, our findings demonstrate that PFASs can mimic AD-like pathophysiology by downregulating skin barrier genes and suppressing the calcitriol–VDR axis in HEKn cells. Moreover, calcitriol may partially reverse PFAS-induced damage by restoring antimicrobial peptide expression and enhancing vitamin D signaling. These insights offer a mechanistic understanding of how environmental pollutants may contribute to skin barrier dysfunction and suggest potential therapeutic avenues for AD based on calcitriol supplementation. While further in vivo validation is required, our findings raise the possibility that targeting the VDR pathway with calcitriol could offer therapeutic value in managing AD symptoms exacerbated by environmental factors such as PFAS exposure.

## 4. Materials and Methods

### 4.1. Cell Culture

Human Epidermal Keratinocyte, neonatal (HEKn) cells were purchased from Gibco™ (Thermo Fisher Scientific, Waltham, MA, USA). HEKn cells were cultured according to the manufacturer’s instructions in EpiLife™ Medium supplemented with Human Keratinocyte Growth Supplement (HKGS, Gibco; containing 0.2% *v/v* bovine pituitary extract, 0.01 μg/mL recombinant human insulin-like growth factor-I, 0.18 μg/mL hydrocortisone, 5 μg/mL bovine transferrin, and 0.2 ng/mL human epidermal growth factor; Gibco) and 60 μM calcium. Cells were maintained in a humidified incubator at 37 °C with 5% CO_2_. All experiments were performed using cells harvested between passages 2 and 6.

### 4.2. MTS Assay

Cell viability was assessed using the MTS assay. HEKn cells were seeded into 96-well plates at a density of 1 × 10^4^ cells per well and allowed to attach for 24 h. Cells were then treated with a range of concentrations of PFNA, PFOA, or a combination of both compounds (Combined PFASs). All PFAS reagents were purchased from Sigma-Aldrich (St. Louis, MO, USA) and dissolved in triple-distilled water. After 24 h of treatment, 20 μL of CellTiter 96^®^ AQueous One Solution Cell Proliferation Assay reagent (Promega, Madison, WI, USA) was added to each well, followed by incubation at 37 °C for 2 h. Absorbance was measured at 490 nm using a microplate reader. Cell viability was calculated by comparing the absorbance values of treated wells to those of the untreated control group. Data were presented as mean ± SD, and all experiments were performed in sextuplicate (*n* = 6).

### 4.3. Cell Morphology and Hoechst 33342/PI Double Staining

To evaluate morphological changes in HEKn cells, microscopic images were acquired following PFAS treatment. Cells were seeded at a density of 2 × 10^5^ cells per well in 6-well plates and allowed to adhere for 24 h before treatment with each PFAS at its respective IC_25_ concentration for 24 h. Phase-contrast images were captured at 10× magnification.

For the assessment of cell death, Hoechst 33342/PI double staining was performed. After PFAS treatment under the same conditions, cells were washed twice with PBS on ice and stained using the Hoechst 33342/PI Double Staining Kit (APE × BIO, Houston, TX, USA), according to the manufacturer’s protocol. Cells were incubated for 30 min on ice in the dark and then washed twice with PBS. Fluorescence images were acquired using a Leica fluorescence microscope equipped with LAS Microscope Software, version 4.3.0. Hoechst 33342 stained all nuclei blue (Excitation: 352 nm/Emission: 461 nm), whereas PI selectively stained the nuclei of membrane-compromised cells red (excitation: 535 nm/emission: 617 nm). Images were captured at 20× magnification. Fluorescence signal quantification and merged image generation were performed using ImageJ software, version 1.53m.

### 4.4. RT-qPCR

HEKn cells were seeded in 6-well plates and cultured for 24 h to allow attachment. Where indicated, cells were induced to differentiate using 1.3 mM CaCl_2_ for 24 h. Subsequently, cells were treated with PFNA, PFOA, or a combination of both, with or without calcitriol (Cayman Chemical) under designated conditions, and harvested at 24 h and 48 h post-treatment. Total RNA was extracted using TRIzol Reagent (Takara Bio Inc., Shiga, Japan) according to the manufacturer’s instructions. RNA concentration and purity were assessed using a NanoDrop™ 2000 spectrophotometer (Thermo Fisher Scientific). Complementary DNA (cDNA) was synthesized using the SuperScript™ IV First-Strand Synthesis System (Invitrogen, Thermo Fisher Scientific, Waltham, MA, USA). RT-qPCR was performed using the HulkMax^®^ Probe qPCR 2X No-ROX Kit (Hulkmax, Precare Gene, Republic of Korea) on the CFX96 Real-Time PCR Detection System (Bio-Rad, Hercules, CA, USA). Each 20 μL reaction mixture comprised 100 ng of cDNA template, 0.5 μM of each forward and reverse primer, and the SYBR Green master mix. Thermal cycling conditions were as follows: initial denaturation at 95 °C for 3 min, followed by 40 cycles of 95 °C for 10 s and 60 °C for 30 s. Melting curve analysis was performed to confirm specific amplification. Relative gene expression levels were calculated using the 2^−ΔΔCt^ method. All results are expressed as mean ± SEM. *GAPDH* was used as the housekeeping gene for normalization. All experiments were conducted in triplicate and included a non-template control (NTC). Primer sequences used in this study are listed in Table 3.

### 4.5. Differentially Expressed Gene (DEG) Analysis

FASTQ files obtained from RNA-seq were processed using CLC Genomics Workbench (version 25.0, QIAGEN, Hilden, Germany). Quality trimming, reference genome mapping to Homo sapiens (hg38), and read counting were conducted within the platform. DEGs were identified using the “Differential Expression for RNA-Seq” module. Cutoff thresholds were set to |log_2_ fold change| > 1 and *p*-value < 0.05.

### 4.6. Ingenuity Pathway Analysis (IPA)

To identify gene sets specifically affected by both PFAS exposure and AD status, DEGs were selected based on comparative DEG analyses. These filtered gene lists were subsequently analyzed using Ingenuity Pathway Analysis (IPA, version 17.6, QIAGEN). Functional analyses included “Diseases and Disorders,” “Molecular and Cellular Functions,” and “Upstream Regulators.” The significance threshold was set to *p*-value < 0.05, and regulatory activity predictions were considered significant at an activation z-score ≥ |1.5|.

### 4.7. Ethical Considerations

Written informed consent was obtained from all participants prior to their inclusion in the study. This research was approved by the Institutional Review Board (IRB) of Samsung Medical Center, Seoul, Korea (IRB approval number: 2016-12-111). Written consent was also obtained from the parents or legal guardians of all participating neonates.

### 4.8. Statistical Analysis

All experiments were performed in at least three independent replicates. Data are presented as mean ± SD or mean ± SEM. Comparisons between two groups were conducted using Student’s *t*-test. Statistical analyses were performed using GraphPad Prism (version 8.0.2, GraphPad Software). Results were considered statistically significant at *p* < 0.05.

## Figures and Tables

**Table 1:** IC values for PFNA, PFOA, and Combined treatment groups in HEKn.

	PFNA	PFOA	Combined
IC10 (μM)	26.0	113.9	22.7
IC25 (μM)	50.9	167.2	40.4
IC50 (μM)	92.3	255.8	69.8

**Table 2:** Cohort group information for DEG analysis.

PFAS Type	Group Name	Exposure Level	Atopic Status	Sample Size (n)	Sex		PFAS Concentration (μg/L)
					Boy	Girl	
PFNA	PFNA_H_A	High	AD	10	8	2	0.797 ± 0.146
	PFNA_H_NA	High	Non-AD	18	12	6	0.767 ± 0.116
	PFNA_L_A	Low	AD	7	5	2	0.394 ± 0.035
	PFNA_L_NA	Low	Non-AD	21	9	12	0.321 ± 0.105
PFOA	PFOA_H_A	High	AD	8	8	0	4.072 ± 0.514
	PFOA_H_NA	High	Non-AD	20	12	8	4.362 ± 1.200
	PFOA_L_A	Low	AD	10 (1 unknown)	6	3	1.432 ± 0.224
	PFOA_L_NA	Low	Non-AD	18	6	12	1.518 ± 0.342

**Table 3:** Primer sequences used for RT-qPCR.

Gene		Sequence (5′ → 3′)	Amplicon Size (Basepair)
*CAMP*	Forward	TGGTGAAGCGGTGTATG	92
	Reverse	CAGGGCAAATCTCTTGTTATC	
*CLDN1*	Forward	CTGTGGCTAAACAGATGTAATG	117
	Reverse	GGGCATCACTGAACAGATA	
*CYP24A1*	Forward	GGCAGAAGATTTGAGGAATATG	97
	Reverse	GTCAAGAGTCCGAGTTGTAA	
*DEFB4A*	Forward	CCATGAGGGTCTTGTATCTC	92
	Reverse	GGTAACAGGATCGCCTATAC	
*FLG*	Forward	CCAGATATGGTTGATGTCTTC	166
	Reverse	GACTGTGCTTTCTGTGC	
*IVL*	Forward	GTGACCCTCTCCCCT	172
	Reverse	CAGTCATGTGCTTTTCCTC	
*LOR*	Forward	TGATGCTACCCGAGGTT	122
	Reverse	TGGGAGGTAGTTGTACAGAA	
*VDR*	Forward	AAGCCACGTTCCTTACTG	122
	Reverse	GTACCTGCTACCCTGTATATTAG	
*GAPDH*	Forward	CAAGGTCATCCCTGAGC	143
	Reverse	CTGCTTCACCACCTTCT	

## Data Availability

The data presented in this study are available upon request from the corresponding author.

## Supplementary Materials

The following supporting information can be downloaded at: https://www.mdpi.com/article/10.3390/ijms26157085/s1.

## Abbreviations

The following abbreviations are used in this manuscript:

AD	Atopic dermatitis
AMP	Antimicrobial peptide
calcitriol	1,25-dihydroxyvitamin D_3_
CAMP	Cathelicidin antimicrobial peptide
CLDN1	Claudin-1
DEFB4A	Defensin beta 4A
DEG	Differential gene expression
EDC	Epidermal differentiation complex
FLG	Filaggrin
FOXO1	Forkhead Box O1
GAPDH	Glyceraldehyde-3-phosphate dehydrogenase
GATA3	GATA binding protein 3
GSTM1	Glutathione S-transferase mu 1
GSTT1	Glutathione S-transferase theta 1
HEKn	Human Epithelial Keratinocyte, neonatal
HKGS	Human Keratinocyte Growth Supplement
IC	Inhibitory concentration
IL6	Interleukin 6
IPA	Ingenuity Pathway Analysis
IVL	Involucrin
JUN	Jun proto-oncogene, AP-1 transcription factor subunit
LOR	Loricrin
PBMC	Peripheral blood mononuclear cell
PFAS	Per- and polyfluroalkyl substances
PFCA	Perfluorocarboxylic acid
PFNA	Perfluorononanoic acid
PFOA	Perfluorooctanoic acid
PFOA	Perfluorooctanesulfonic acid
PI	Propidium iodide
PM	Particulate matter
PPARGC1	Peroxisome proliferator-activated receptor gamma coactivator 1-alpha
RELA	RELA proto-oncogene, NF-κB subunit
STAT3	Signal transducer and activator of transcription 3
TEWL	Transepidermal water loss
VDR	Vitamin D receptor
VOC	Volatile organic compound
ZO	Zonula occludens

## References

[1] Bonamonte D., Filoni A., Vestita M., Romita P., Foti C., Angelini G. (2019). The role of the environmental risk factors in the pathogenesis and clinical outcome of atopic dermatitis. BioMed Res. Int..

[2] Kantor R., Silverberg J.I. (2017). Environmental risk factors and their role in the management of atopic dermatitis. Expert Rev. Clin. Immunol..

[3] Stefanovic N., Irvine A.D., Flohr C. (2021). The role of the environment and exposome in atopic dermatitis. Curr. Treat. Options Allergy.

[4] Lee W., Chaudhary F., Agrawal D.K. (2024). Environmental Influences on Atopic Eczema. J. Environ. Sci. Public Health.

[5] Lai A., Owens K., Patel S., Nicholas M. (2023). The impact of air pollution on atopic dermatitis. Curr. Allergy Asthma Rep..

[6] Umehara Y., Trujillo-Paez J.V., Yue H., Peng G., Nguyen H.L.T., Okumura K., Ogawa H., Niyonsaba F. (2023). Calcitriol, an active form of vitamin D3, mitigates skin barrier dysfunction in atopic dermatitis NC/Nga mice. Int. J. Mol. Sci..

[7] Ramanathan Jr M., Lee W.-K., Spannhake E.W., Lane A.P. (2008). Th2 cytokines associated with chronic rhinosinusitis with polyps down-regulate the antimicrobial immune function of human sinonasal epithelial cells. Am. J. Rhinol..

[8] Kotthoff M., Müller J., Jürling H., Schlummer M., Fiedler D. (2015). Perfluoroalkyl and polyfluoroalkyl substances in consumer products. Environ. Sci. Pollut. Res..

[9] Chen Q., Huang R., Hua L., Guo Y., Huang L., Zhao Y., Wang X., Zhang J. (2018). Prenatal exposure to perfluoroalkyl and polyfluoroalkyl substances and childhood atopic dermatitis: A prospective birth cohort study. Environ. Health.

[10] Rosato I., Bonato T., Fletcher T., Batzella E., Canova C. (2024). Estimation of per-and polyfluoroalkyl substances (PFAS) half-lives in human studies: A systematic review and meta-analysis. Environ. Res..

[11] Skinner J.P., Raderstorf A., Rittmann B.E., Delgado A.G. (2025). Biotransforming the “Forever Chemicals”: Trends and Insights from Microbiological Studies on PFAS. Environ. Sci. Technol..

[12] Figuière R., Miaz L.T., Savvidou E., Cousins I.T. (2025). An Overview of Potential Alternatives for the Multiple Uses of Per-and Polyfluoroalkyl Substances. Environ. Sci. Technol..

[13] Eriksen K.T., Raaschou-Nielsen O., Sørensen M., Roursgaard M., Loft S., Møller P. (2010). Genotoxic potential of the perfluorinated chemicals PFOA, PFOS, PFBS, PFNA and PFHxA in human HepG2 cells. Mutat. Res./Genet. Toxicol. Environ. Mutagen..

[14] Hatem G., Faria A.M., Pinto M.B., Salamova A., Teixeira J.P., Costa C., Madureira J. (2025). Exposure to per-and poly-fluoroalkyl substances and respiratory and skin effects in children and adolescents: A systematic review and meta-analysis. J. Hazard. Mater..

[15] Wen H.-J., Wang S.-L., Chuang Y.-C., Chen P.-C., Guo Y.L. (2019). Prenatal perfluorooctanoic acid exposure is associated with early onset atopic dermatitis in 5-year-old children. Chemosphere.

[16] Wen H.-J., Wang S.-L., Chen P.-C., Guo Y.L. (2019). Prenatal perfluorooctanoic acid exposure and glutathione s-transferase T1/M1 genotypes and their association with atopic dermatitis at 2 years of age. PLoS ONE.

[17] Belloc F., Dumain P., Boisseau M.R., Jalloustre C., Reiffers J., Bernard P., Lacombe F. (1994). A flow cytometric method using Hoechst 33342 and propidium iodide for simultaneous cell cycle analysis and apoptosis determination in unfixed cells. Cytom. J. Int. Soc. Anal. Cytol..

[18] Barthe M., Clerbaux L.-A., Thénot J.-P., Braud V.M., Osman-Ponchet H. (2024). Systematic characterization of the barrier function of diverse ex vivo models of damaged human skin. Front. Med..

[19] Bao L., Zhang H., Chan L.S. (2013). The involvement of the JAK-STAT signaling pathway in chronic inflammatory skin disease atopic dermatitis. Jak-Stat.

[20] Diago C.A.A., García-Unzueta M.T., Fariñas M.d.C., Amado J.A. (2016). Calcitriol-modulated human antibiotics: New pathophysiological aspects of vitamin D. Endocrinol. Y Nutr. (Engl. Ed.).

[21] White J.H. (2022). Emerging roles of vitamin D-induced antimicrobial peptides in antiviral innate immunity. Nutrients.

[22] Glüge J., Scheringer M., Cousins I.T., DeWitt J.C., Goldenman G., Herzke D., Lohmann R., Ng C.A., Trier X., Wang Z. (2020). An overview of the uses of per-and polyfluoroalkyl substances (PFAS). Environ. Sci. Process. Impacts.

[23] Brennan N.M., Evans A.T., Fritz M.K., Peak S.A., von Holst H.E. (2021). Trends in the regulation of per-and polyfluoroalkyl substances (PFAS): A scoping review. Int. J. Environ. Res. Public Health.

[24] Li J., Sun J., Li P. (2022). Exposure routes, bioaccumulation and toxic effects of per-and polyfluoroalkyl substances (PFASs) on plants: A critical review. Environ. Int..

[25] Tukker A.M., Bouwman L.M.S., van Kleef R.G.D.M., Hendriks H.S., Legler J., Westerink R.H.S. (2020). Perfluorooctane sulfonate (PFOS) and perfluorooctanoate (PFOA) acutely affect human α1β2γ2L GABAA receptor and spontaneous neuronal network function in vitro. Sci. Rep..

[26] Frølunde A.S., Vestergaard C., Deleuran M. (2022). Skin Barrier Abnormalities in Atopic Dermatitis. Curr. Treat. Options Allergy.

[27] Leung D.Y., Berdyshev E., Goleva E. (2020). Cutaneous barrier dysfunction in allergic diseases. J. Allergy Clin. Immunol..

[28] Katsarou S., Makris M., Vakirlis E., Gregoriou S. (2023). The role of tight junctions in atopic dermatitis: A systematic review. J. Clin. Med..

[29] Hashimoto-Hachiya A., Tsuji G., Murai M., Yan X., Furue M. (2018). Upregulation of FLG, LOR, and IVL expression by Rhodiola crenulata root extract via aryl hydrocarbon receptor: Differential involvement of OVOL1. Int. J. Mol. Sci..

[30] Evich M.G., Davis M.J., McCord J.P., Acrey B., Awkerman J.A., Knappe D.R., Lindstrom A.B., Speth T.F., Tebes-Stevens C., Strynar M.J. (2022). Per-and polyfluoroalkyl substances in the environment. Science.

[31] Buck R.C., Franklin J., Berger U., Conder J.M., Cousins I.T., De Voogt P., Jensen A.A., Kannan K., Mabury S.A., van Leeuwen S.P. (2011). Perfluoroalkyl and polyfluoroalkyl substances in the environment: Terminology, classification, and origins. Integr. Environ. Assess. Manag..

[32] Fenton S.E., Ducatman A., Boobis A., DeWitt J.C., Lau C., Ng C., Smith J.S., Roberts S.M. (2021). Per-and polyfluoroalkyl substance toxicity and human health review: Current state of knowledge and strategies for informing future research. Environ. Toxicol. Chem..

[33] Lu R., Peng Z., Lian P., Wazir J., Gu C., Ma C., Wei L., Li L., Pu W., Liu J. (2023). Vitamin D attenuates DNCB-induced atopic dermatitis-like skin lesions by inhibiting immune response and restoring skin barrier function. Int. Immunopharmacol..

[34] Hong S., Oh Y., Jung M., Lee S., Jeon H., Cho M.Y., Lee S., Choi E. (2010). Topical calcitriol restores the impairment of epidermal permeability and antimicrobial barriers induced by corticosteroids. Br. J. Dermatol..

[35] Singam E.R.A., Durkin K.A., La Merrill M.A., Furlow J.D., Wang J.-C., Smith M.T. (2023). The vitamin D receptor as a potential target for the toxic effects of per-and polyfluoroalkyl substances (PFASs): An in-silico study. Environ. Res..

[36] Muindi J.R., Yu W.-D., Ma Y., Engler K.L., Kong R.-X., Trump D.L., Johnson C.S. (2010). CYP24A1 inhibition enhances the antitumor activity of calcitriol. Endocrinology.

[37] Suwanchote S., Waitayangkoon P., Chancheewa B., Inthanachai T., Niwetbowornchai N., Edwards S.W., Virakul S., Thammahong A., Kiatsurayanon C., Rerknimitr P. (2022). Role of antimicrobial peptides in atopic dermatitis. Int. J. Dermatol..

[38] Gonzalez-Curiel I., Trujillo V., Montoya-Rosales A., Rincon K., Rivas-Calderon B., deHaro-Acosta J., Marin-Luevano P., Lozano-Lopez D., Enciso-Moreno J.A., Rivas-Santiago B. (2014). 1, 25-dihydroxyvitamin D3 induces LL-37 and HBD-2 production in keratinocytes from diabetic foot ulcers promoting wound healing: An in vitro model. PLoS ONE.

[39] Roy S., Danasekaran K., Moran J., O’Brien K., Dakshanamurthy S. (2024). Comprehensive Analysis and Large-Scale Screening of Binding Interactions Between PFAS and Their Mixtures with Nuclear Receptors. Preprints.

[40] Wang T.-T., Nestel F.P., Bourdeau V., Nagai Y., Wang Q., Liao J., Tavera-Mendoza L., Lin R., Hanrahan J.W., Mader S. (2004). Cutting edge: 1, 25-dihydroxyvitamin D3 is a direct inducer of antimicrobial peptide gene expression. J. Immunol..

